# Turnbull on Diseases of the Stomach
†A Practical Treatise on Disorders of the Stomach with Fermentation. By James Turnbull, M.D. London: Churchill. 1856.


**Published:** 1856-10

**Authors:** 


					TURN BULL ON DISEASES OF THE STOMACH.f
Dr. Txjrnbull's Treatise is a useful work. The chapters
on the Chemistry of Fermentation, on Fermentive Disorder,
and on Diet, are clearly and carefully written. The author
propounds no particular theory or new fact, but attempts
a comprehensive review of his subject, in which task he
succeeds well.
+ A Practical Treatise on Disorders of the Stomach with Fermentation.
By James Turnbull, M.D. London : Churchill. 1856.

				

